# 14-3-3ζ: an optimal housekeeping protein for western blot analysis in swine rotator cuff tendon studies

**DOI:** 10.1007/s11010-025-05255-6

**Published:** 2025-03-23

**Authors:** Resmi Rajalekshmi, Vikrant Rai, Devendra K. Agrawal

**Affiliations:** Department of Translational Research, College of Osteopathic Medicine of the Pacific, Western University of Health Sciences, 309 E. Second Street, Pomona, CA 91766 USA

**Keywords:** 14-3-3ζ, Housekeeping gene, Hyperlipidemia, Rotator cuff tendon, Swine model, Western blot analysis, YWHAZ

## Abstract

Healthy biomechanics of the shoulder involving rotator cuff muscles and rotator cuff tendon (RCT) is pivotal for joint stability, yet co-morbid conditions like hyperlipidemia and hyperglycemia can lead to degenerative changes jeopardizing tendon integrity. A change in protein expression, the functional moiety for molecular events, may result in altered healing of RCT and prolonged morbidity. Expression and activity of proteins are critical while investigating the underlying molecular and cellular changes involved in tendinopathy. While investigating the changes in the protein expression of various inflammatory mediators, we observed that the Western Blot bands for commonly used housekeeping genes (GAPDH, β-actin, and α-tubulin) were not uniform in different tendon samples. Therefore, we investigated for an optimal housekeeping gene for Western blot analysis in swine RCT under normal and hyperlipidemic conditions, as this is essential for accurate normalization of protein expression. The study evaluated several housekeeping genes—GAPDH, beta-actin, alpha and beta-tubulin, Ubiquitin C, Cyclophilin A, TATA-box binding protein, and 14-3-3ζ—to ensure robust normalization across experimental setups. The results revealed that the protein expression of 14-3-3ζ was uniform in all samples, thereby validating its suitability as a stable housekeeping protein. The findings are important while studying the RCT pathology in a clinically relevant animal model, like swine, which mimics human RCT and provides translationally significant findings. Thus, the 14-3-3ζ protein will be an ideal housekeeping gene in the design of experiments utilizing musculoskeletal tissues.

## Introduction

The rotator cuff tendon (RCT) is crucial in maintaining stability in the shoulder joint and enabling precise movements necessary for everyday tasks. However, conditions such as rotator cuff tears and rotator cuff injury can severely impact mobility and overall quality of life in humans and animals [1]. Recent studies have increasingly delved into the effects of metabolic disorders like hyperlipidemia on musculoskeletal health [2–4]. Elevated lipid levels in the bloodstream, a characteristic of hyperlipidemia, can have detrimental effects on tendon physiology, leading to degenerative changes that compromise the structure, mechanical properties, and overall resilience of tendons [5]. Thus, understanding the molecular mechanisms underlying these changes is crucial for devising targeted therapeutic interventions.

Swine models, known for their physiological similarities with humans and comparable tendon structure and biomechanics, provide an excellent translational platform for studying rotator cuff tendon responses [6]. Leveraging swine models enables researchers to understand molecular responses to hyperlipidemic conditions in the rotator cuff tendon, potentially revealing biomarkers and therapeutic targets relevant to human tendon disorders.

Western blot analysis, a powerful molecular biology tool, is used to quantify protein expression levels with high specificity and sensitivity [7]. The accuracy of Western blotting hinges on the selection of an appropriate housekeeping gene, which serves as an internal control for normalizing target protein expression [8]. However, finding a reliable housekeeping gene that maintains stable expression across diverse experimental conditions, including those influenced by hyperlipidemia, remains a significant challenge. Commonly used housekeeping genes such as glyceraldehyde-3-phosphate dehydrogenase (GAPDH) and beta-actin have been widely utilized in mRNA expression studies and protein analysis [9]. However, their stability under various experimental conditions may vary significantly due to factors such as tissue type, species differences, experimental protocols, and reagent variability [10]. Therefore, a thorough evaluation of candidate housekeeping genes is essential before their use in any experimental design. While assessing the protein expression of various inflammatory proteins using RCT tissues from various experimental groups we observed nonuniform pattern of GAPDH, β-actin, and α-tubulin in the tendon tissue only and not in other soft tissues.

Thus, we designed experiments to investigate an optimal housekeeping protein with uniform pattern in Yucatan miniswine rotator cuff tendon tissues under non-hyperlipidemic and hyperlipidemic conditions from an ongoing study to examine the effect of TLR4 antagonist, TAK-242, which inhibits TLR-4 signaling by binding to TLR4, disrupting interactions with adaptors, and reducing proinflammatory effects [11]. The housekeeping genes of interest included GAPDH, beta-actin, alpha and beta-tubulin, ubiquitin C (UBC), Peptidylprolyl Isomerase A (PPIA, also known as Cyclophilin A), TATA box binding protein (TBP), and tyrosine 3-monooxygenase/tryptophan 5-monooxygenase activation protein zeta (14-3-3ζ). The findings of this study provide an evidence of an optimal housekeeping protein for accurate and consistent analyses of protein expression, particularly in tendons, thereby advancing our understanding of tendon biology and pathology.

## Materials and methods

### Animal model and tissue collection

The protocol to conduct the studies were approved by the IACUC of Western University of Health Sciences, Pomona, California (Protocols #R19IACUC026 and #R20IACUC038). RCT tissues collected from four to seven-month-old Yucatan miniswine, each weighing between 20–30 kg, being used for other studies [11] in the lab were obtained from Premier Bio-resources (Ramona, CA, USA). Female pigs were selected for this study due to their lower aggression levels and ease of handling. Male pigs available commercially are typically castrated for safety, which induces hormonal changes that could confound the study results. The miniswine were housed at the Western University of Health Science animal facility at Pomona, CA, under a 12-h light/dark cycle with temperatures maintained between 72°F and 74°F. The swine in the non-hyperlipidemic group were fed with Mini-Pig Grower Diet (Test Diet #5801). For the hyperlipidemic condition, they were fed with an experimental high-cholesterol diet (D17012601) composed of 51% carbohydrates, 20% protein, 10% fat, and 4% cholesterol. Water was available ad libitum. For this study, rotator cuff tendon tissues (RCTs) were collected from 18 animals: The non-hyperlipidemic group included 10 animals (5 treated with ethanol and 5 with TAK-242 (dissolved in 30% ethanol), while the hyperlipidemic group consisted of 8 animals (4 treated with ethanol and 4 with TAK-242). Following euthanasia after 40 weeks on the control or high cholesterol diet, RCTs were collected from swine and stored at − 80 °C until further use.

### Western blot

Approximately 200 mg of tendon tissue from each sample was used for total protein isolation. Tissue fragments were homogenized in 1 mL of RIPA Lysis and Extraction Buffer (PI89901, ThermoFisher Scientific, Waltham, MA, USA), supplemented with a protease inhibitor cocktail (Pierce Protease Inhibitor Mini Tablets, A32953, ThermoFisher Scientific, Waltham, MA, USA), using a tissue disruptor (PowerGen 125, ThermoFisher Scientific, Waltham, MA, USA). Following complete dissociation, samples were incubated overnight at 4 °C with shaking and then centrifuged at 4 °C for 30 min to remove insoluble fragments. The supernatants were transferred to new Eppendorf tubes, and total protein concentrations were determined using the Bradford method [12] with the Bio-Rad Protein Assay Kit II (5,000,002, Bio-Rad Laboratories, Hercules, CA, USA). A total of 25 µg of protein was loaded onto SDS gels (4–15% Mini-PROTEAN TGX Precast Protein Gels, 4,561,084, Bio-Rad Laboratories, Hercules, CA, USA) and transferred to PVDF membranes (1,620,177, Bio-Rad Laboratories, Hercules, CA, USA) following standard procedures. The equal loading o the protein in each well was assessed and confirmed using Ponceau Red staining (P7170, Millipore Sigma, Burlington, MA, USA) (data not shown). The non-specific proteins were then blocked for 1 h at room temperature with 5% skimmed milk (1,706,404, Bio-Rad Laboratories, Hercules, CA, USA) in TBST (1 × Tris Buffered Saline (TBS), 50–489-119, ThermoFisher Scientific, Waltham, MA, USA, supplemented with 0.1% Tween20, P1379, Millipore Sigma, Burlington, MA, USA). Subsequently, membranes were incubated with primary antibodies (Table 1) in the blocking solution overnight at 4 °C with gentle agitation. After incubation, the membranes were washed three times for 5 min each in 1xTBST and were incubated with appropriate secondary antibodies (Table 1) for 1 h at room temperature with gentle agitation. Finally, the membranes were washed, and signals were developed using Pierce ECL Western Blotting Substrate (32,106, ThermoFisher Scientific, Waltham, MA, USA). Images were captured using a ChemiDoc XRS + System (Bio-Rad Laboratories, Hercules, CA, USA) with an exposure time of 200 s and processed with Fiji Image J Software (version 1.54 J, NIH, USA).

**Table 1:** Information on the source and catalogue number of the primary and secondary antibodies and their dilution factors used in this study for Western blot

Antibody	Supplier	Catalog	Dilution
Primary antibodies			
GAPDH	Proteintech	60,004-1-Ig	1:50,000
Beta actin	Abcam	ab8226	1:1000
Alpha tubulin	Abcam	ab80779	1:1000
Beta tubulin	Abcam	ab11315	1:1000
UBC	Mybiosource.com	MBS7113494	1:2000
PPIA	Mybiosource.com	MBS3210325	1 μg/ml
TBP	Abcam	ab300656	1:1000
14-3-3ζ	Mybiosource.com	MBS9601925	1:1000
Secondary antibodies			
Anti-mouse	Novus biologicals	NB7544	1:3000
Anti-rabbit	Thermofisher scientific	A16023	1:2000

### Statistics

Data were analyzed using GraphPad Prism 10 for Windows (version 10.1.1) and presented as mean ± standard deviation. The Shapiro–Wilk test was used to assess the normality of the data. Comparisons between the non-hyperlipidemic and hyperlipidemic groups for each swine were conducted using the Student’s *t*-test.

## Results

### Validation of loading control for western blot

**Fig. 1:**
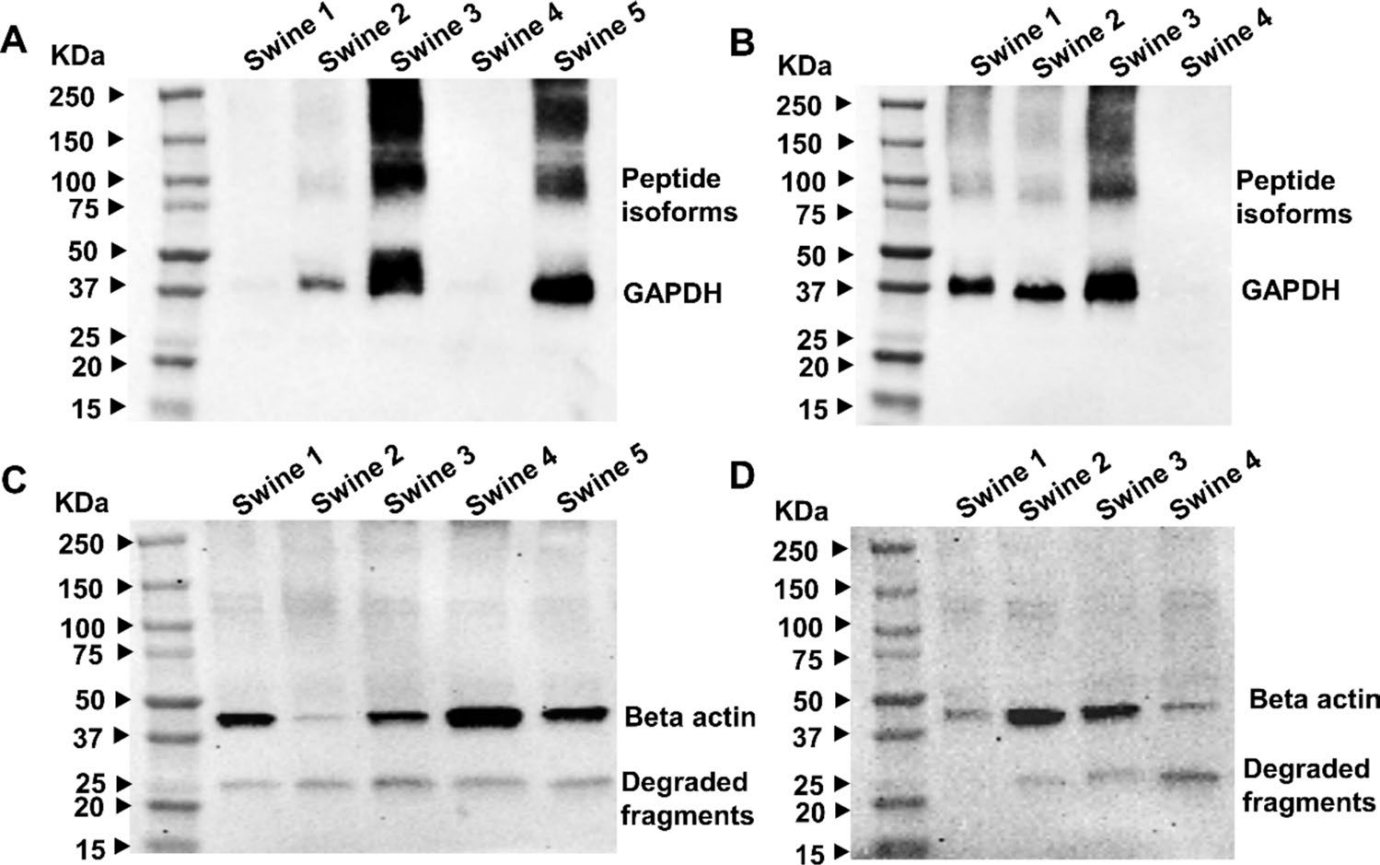
Western Blot Analysis of GAPDH and beta-actin in individual non-hyperlipidemic and hyperlipidemic swine. A total of 25 µg of protein in each lane was loaded onto SDS gels. GAPDH expression (37 kDa) in non-hyperlipidemic (**A**) and hyperlipidemic (**B**) swine shows variable band intensity. Beta-actin (42 kDa) is similarly variable in non-hyperlipidemic (**C**) and hyperlipidemic (D) groups. Both GAPDH and beta-actin display nonuniform expression, indicating sample variability. N = 5 swine tissues for non-hyperlipidemic and N = 4 swine tissues for hyperlipidemic experimental groups

The Western blot images of GAPDH (Fig. 1A, B) illustrate variations in expression levels (nonuniform bands) in both non-hyperlipidemic and hyperlipidemic swine. These images depict distinct bands at the expected molecular weight of 37 kDa, corresponding to the full-length GAPDH protein. Furthermore, faint bands are evident in the range of 75 to 100 kDa, suggesting the presence of short peptide isoforms resulting from post-translational modifications [13].

The 42 kDa beta-actin band was observed in both groups of animals (Fig. 1C, D), but the expressions were not consistent. Additionally, bands for beta-actin around 25 kDa were also observed. These may be degraded fragments or unknown isoforms smaller than the wild protein, possibly resulting from mechanisms like translation initiated from a downstream start codon [14].

**Fig. 2:**
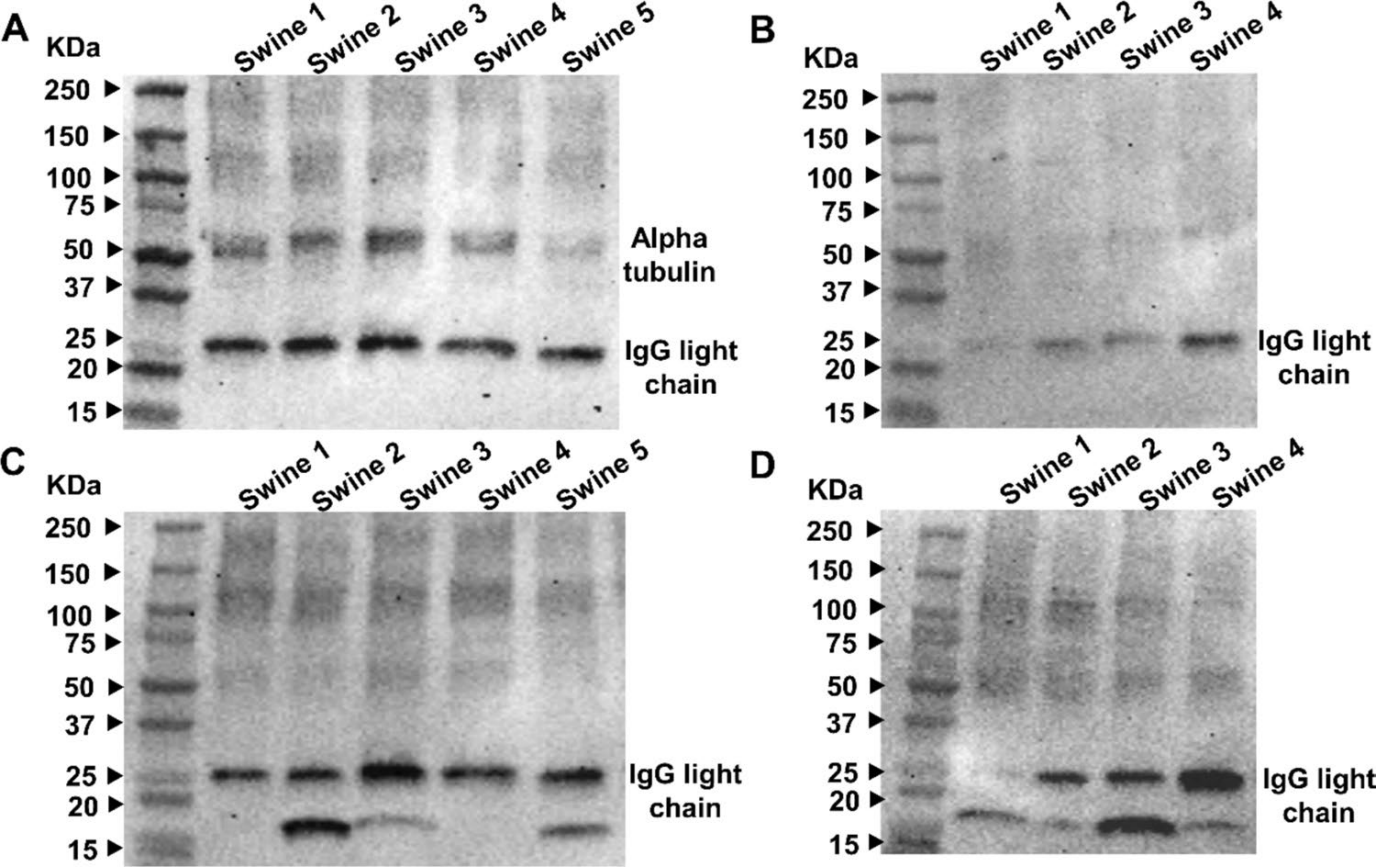
Western blot analysis of alpha and beta tubulin in individual swine rotator cuff tendon tissues. A total of 25 µg of protein in each lane was loaded onto SDS gels. **A** Alpha tubulin expression in non-hyperlipidemic samples; **B** Alpha tubulin expression in hyperlipidemic samples. **C** Beta tubulin expression in non-hyperlipidemic samples; **D** Beta tubulin expression in hyperlipidemic samples. This analysis illustrates the differences in the expression levels between the two conditions. N = 5 swine tissues for non-hyperlipidemic and N = 4 swine tissues for hyperlipidemic groups

In Fig. 2A, 55 kDa bands, corresponding to the expected molecular weight of alpha tubulin [15], were present in non-hyperlipidemic swine tendon tissues, but absent in the hyperlipidemic swine tendon tissues. Additionally, a 25 kDa band, corresponding to the IgG light chain, was observed. This band was noted in the antibody testing data from select suppliers, such as antibodies.com for the product Anti-alpha Tubulin Antibody [TU-01] (A86726). The intensity of this 25 kDa band was variable across both animal groups and decreased in hyperlipidemic animals, most likely because of hyperlipidemia. However, the effect of hyperlipidemia on protein expression and the underlying mechanism warrants in-depth studies.

Regarding beta tubulin (Fig. 2C, D), the expected 52 kDa band [16] was absent in both animal groups’ tendons. Instead, a 25 kDa band was observed in both groups. This band has been reported by the antibody supplier Proteintech as a light chain for the product Beta Tubulin Polyclonal Antibody (Cat No. 10068-1-AP), and by Antibodies.com for the product Anti-beta III Tubulin Antibody [TUBB3/3731] (A248107) when the protein is reduced. The intensity of these bands was non-uniform across both animal groups, and in the hyperlipidemic group, this band was completely absent in swine 1. Another band observed for beta-tubulin was around 18 kDa for swine 2, 3, and 5 in the non-hyperlipidemic group and all swine in the hyperlipidemic group. These bands were reported for the product AB9354 from Sigma-Aldrich (Anti-Beta III Tubulin Antibody), although no explanations were provided for this observation. According to the data reported for the product ab18251 from Abcam (Anti-alpha Tubulin Antibody—Microtubule Marker), the lower molecular weight bands are attributed to cross-reactivity of the antibody. Therefore, it is likely that the 18 kDa bands observed for beta-tubulin are also due to antibody cross-reactivity.

**Fig. 3:**
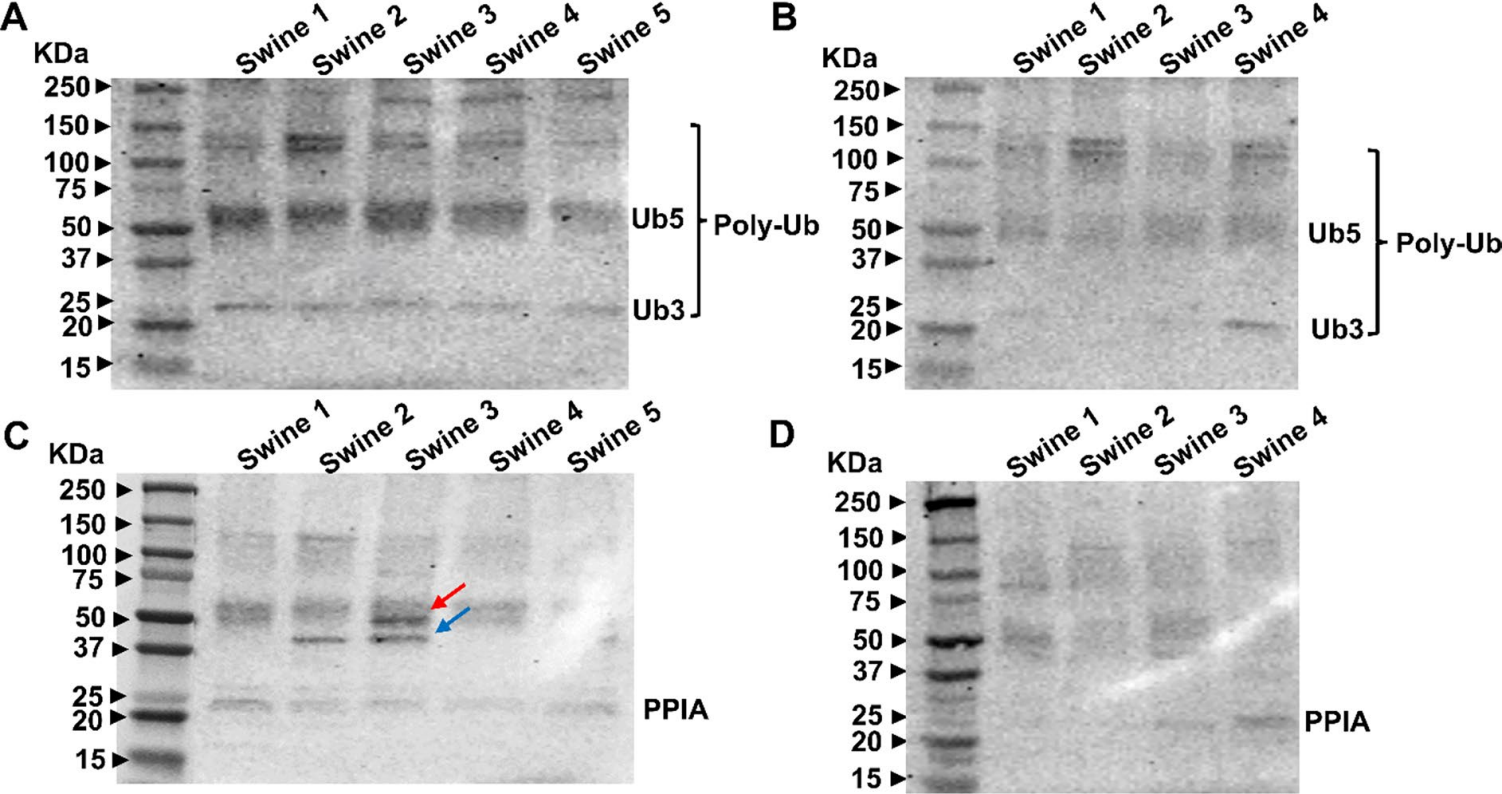
Western blot analysis of UBC and PPIA in individual swine RCT tissues. A total of 25 µg of protein in each lane was loaded onto SDS gels. **A** UBC expression in non-hyperlipidemic samples, **B** UBC expression in hyperlipidemic samples, showing variability between conditions. **C** PPIA expression in non-hyperlipidemic and hyperlipidemic samples respectively. The red arrow indicates the 47 kDa band and the blue arrow indicates the 37 kDa band, highlighting inconsistent expression across both groups. N = 5 swine tissues for non-hyperlipidemic, N = 4 swine tissues for hyperlipidemic

The predicted molecular weight for UBC is 78 kDa, while antibody suppliers report the observed molecular weight as 12–55 kDa (Abcam, Aviva, etc.). In swine rotator cuff tendon tissues, bands at 25, 60, and 140 kDa were observed, corresponding to different lengths of ubiquitin, including ubiquitin dimer (Ub2) and ubiquitin pentamer (Ub5) [17, 18].

as depicted in Fig. 3A, B. The band above 75 kDa represents higher molecular weight ubiquitin species documented in the literature [19]. Collectively, these bands ranging from 25–150 kDa are referred to as polyubiquitin chains (Poly-Ub) [20]. Importantly, these bands were present in non-hyperlipidemic animals but notably reduced in hyperlipidemic swine tendons.

For PPIA, the observed molecular weight in Fig. 3C, D was approximately 20 kDa [21]. These bands, although faint, were consistently observed in all non-hyperlipidemic swine RCT samples. In contrast, in hyperlipidemic swine RCT, these bands were visible only in swine 3 and 4. Additional bands observed around  ~ 37 kDa (blue arrow) in non-hyperlipidemic swine 2 and 3, and  ~ 47 kDa (red arrow) in Swine 3, were not previously reported in the literature. The antibody supplier Sigma-Aldrich indicates a band at 37 kDa for their product SAB2101855 (Anti-PPIA antibody produced in rabbit), without providing further details. These bands were not visible in hyperlipidemic animals.

**Fig. 4:**
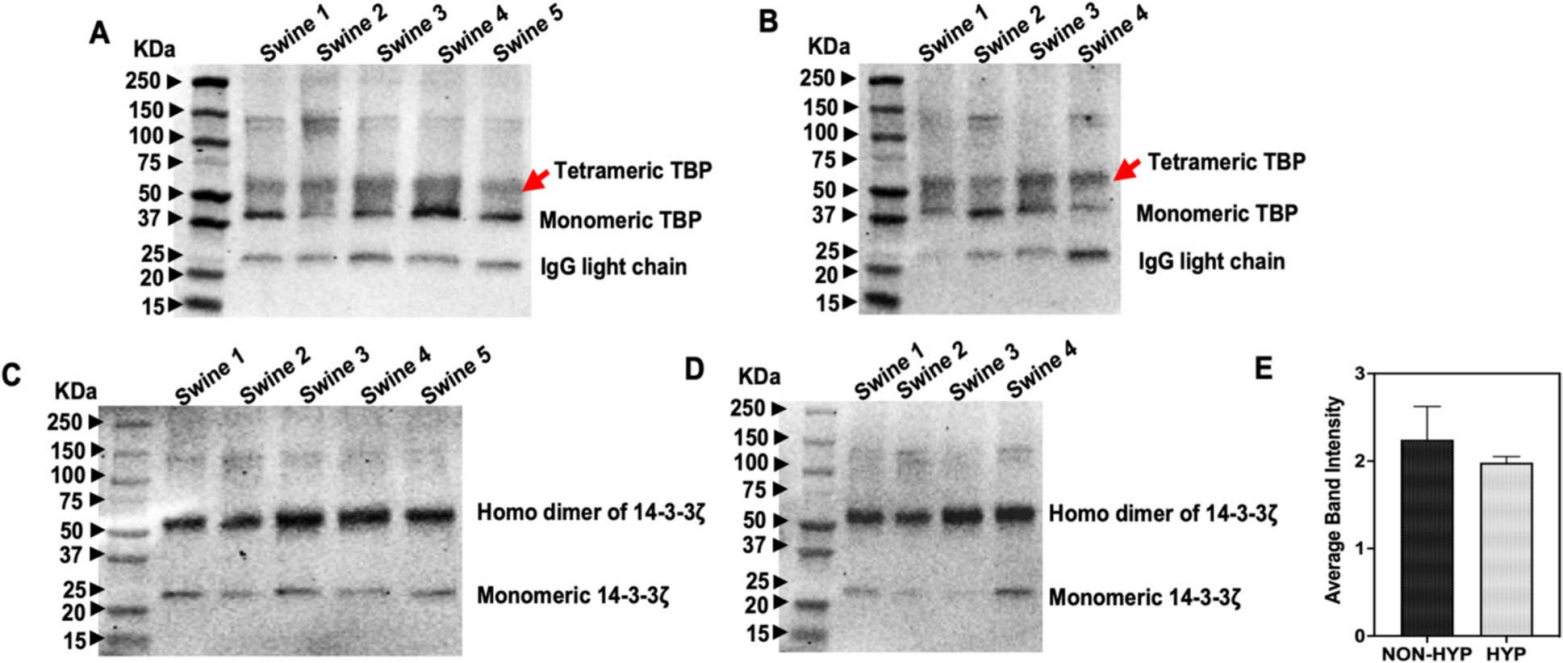
Western blot analysis of TATA box binding protein (TBP) and 14-3-3ζ in individual swine RCT tissues. A total of 25 µg of protein in each lane was loaded onto SDS gels. **A**, **B** TBP expression in non-hyperlipidemic and hyperlipidemic samples respectively; red arrow indicates the 55 kDa tetrameric form of TBP. **C**, **D** 14-3-3ζ expression in non-hyperlipidemic and hyperlipidemic samples respectively, showing consistent expression across both groups. **E** Densitometric analysis of 14-3-3ζ, with average band intensity normalized to the background. NON-HYP: Non-Hyperlipidemic; HYP: Hyperlipidemic. N = 5 swine tissues for non-hyperlipidemic, N = 4 swine tissues for hyperlipidemic groups

In Fig. 4A, B, a prominent band at 38 kDa (monomeric TBP) was consistently observed in both non-hyperlipidemic and hyperlipidemic RCT groups, though expression levels varied. Additionally, a 150 kDa band, identified as the tetrameric form of TBP [22], showed non-uniform expression across animals, notably absent in Swine 1 and 4 of the hyperlipidemic group. A band around 55 kDa (red arrow) was also observed, consistent with the reported molecular weight of the TBP antibody product (Anti-TATA binding protein TBP antibody—Nuclear Loading Control and ChIP Grade, ab63766), while a band at 25 kDa represented the IgG light chain common to other antibodies.

The monomeric form of 14-3-3ζ, observed at 28 kDa, showed non-uniform expression across non-hyperlipidemic swine RCT animals, decreasing in the hyperlipidemic group. Interestingly, a consistently expressed band at 56 kDa in both groups was identified as the covalently crosslinked homodimer of 14-3-3ζ [23]. The uniform bands at 56 KDa across all swine samples, regardless of disease condition, suggest that 14–3-3ζ is a robust candidate for a housekeeping protein in swine RCT studies, as confirmed by densitometric analysis, which showed no significant differences between the groups (Fig. 4C–E).

**Fig. 5:**
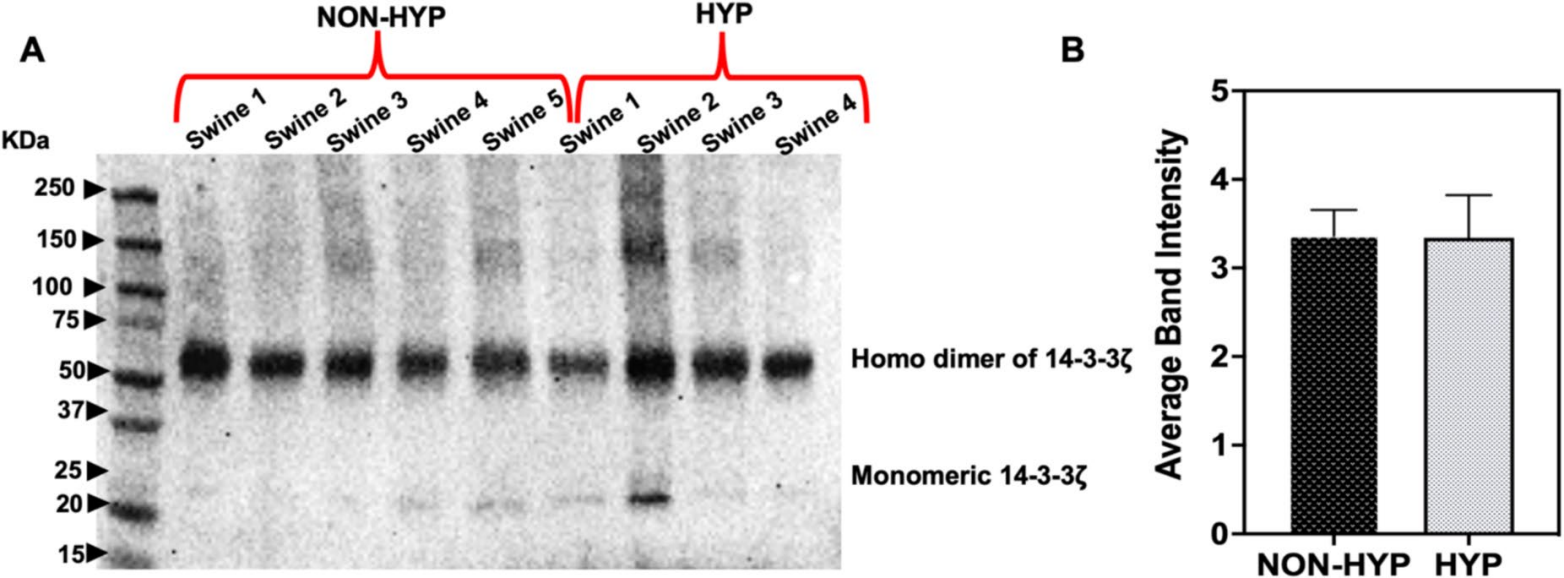
A total of 25 µg of protein in each lane was loaded onto SDS gels. **A** Western blot analysis of 14-3-3ζ in TAK-242-treated swine, showing uniform band intensity. **B** Densitometric analysis of 14-3-3ζ, with average band intensity normalized to the background. NON-HYP: Non-HyperLipidemic; HYP: hyperlipidemic. N = 5 swine tissues for non-hyperlipidemic, N = 4 swine tissues for hyperlipidemic groups

To investigate the impact of treatment conditions on 14-3-3ζ expression, Western blot analysis was performed on non-hyperlipidemic and hyperlipidemic swine treated with TAK 242 (protein expression for these swine is not included in the blots included in Fig. 1–4). Results from TAK-242 treated swine (Fig. 5A, B) indicated that treatment did not alter 14-3-3ζ expression in either animal group, reinforcing its suitability as a stable housekeeping protein for western blot analyses in RCT studies.

## Discussion

The accurate selection of housekeeping genes is essential for reliable Western blot analysis, particularly in studies investigating pathological conditions such as hyperlipidemia. This study evaluated several commonly used housekeeping proteins in swine rotator cuff tendon (RCT) tissues under normal and hyperlipidemic conditions, revealing significant variability in their expression. These findings underscore the importance of selecting appropriate internal controls for accurate protein quantification, especially in disease models.

GAPDH, widely used due to its role in glycolysis and presumed stable expression across tissues [24], showed inconsistent band intensities in both hyperlipidemic and non-hyperlipidemic RCT tissues. This variability, potentially arising from post-translational modifications, was further supported by the presence of faint higher molecular weight bands. Similarly, beta-actin, a cytoskeletal protein often used as a loading control [25], exhibited additional bands, likely representing degradation products or smaller isoforms. Both GAPDH and beta-actin, therefore, appear unreliable as internal controls in hyperlipidemic conditions due to altered expression patterns.

Alpha- and beta-tubulin, essential for cell shape and intracellular transport, also posed challenges [26]. Alpha-tubulin was absent in hyperlipidemic tissues but consistently expressed in non-hyperlipidemic samples. Beta-tubulin, instead of the expected 52 kDa band, displayed lower molecular weight bands (18 kDa and 25 kDa), likely due to antibody cross-reactivity. These findings suggest that tubulins are not suitable as housekeeping genes under hyperlipidemic conditions.

Ubiquitin C (UBC), a key regulator of protein degradation through ubiquitination, showed bands corresponding to different lengths of ubiquitin chains, such as dimers and pentamers [27, 28]. However, hyperlipidemic tendons exhibited a significant reduction in these polyubiquitin chains, indicating disrupted ubiquitin homeostasis. This variability renders UBC an unreliable internal control in hyperlipidemic tissues.

Similarly, Cyclophilin A (CyPA), also known as PPIA, is a widely expressed immunophilin protein with peptidyl-prolyl isomerase activity. It can be secreted in response to inflammatory stimuli such as hypoxia, infection, and oxidative stress [29]. PPIA plays critical roles in protein folding, trafficking, cell activation, and chemotaxis, and has implications in various diseases and aging [30]. Its inconsistent expression of PPIA, an immunophilin with peptidyl-prolyl isomerase activity, raises concerns about its reliability. The appearance of additional, previously unreported bands further highlights its instability in hyperlipidemic tissues.

TATA-binding protein (TBP) which is essential for initiating transcription by binding to the TATA box in promoter regions, helping recruit RNA polymerase II and other factors. It is expressed in all cell types and is crucial for basic cellular functions [31, 32]. However, TBP exhibited variability in this study, particularly in its tetrameric form, which was absent in some hyperlipidemic animals, suggesting altered transcriptional activity due to the disease.

In contrast, 14-3-3ζ emerged as the most consistent housekeeping protein. This conserved protein, involved in critical cellular processes such as signal transduction, apoptosis, and cell cycle regulation, was stably expressed across both normal and hyperlipidemic conditions [33]. Densitometric analysis confirmed no significant differences in expression levels between groups, establishing 14-3-3ζ as a robust internal control. Additionally, previous studies have optimized 14-3-3 zeta as a housekeeping gene for RT-PCR in tendon cells, further supporting its reliability [34].

To assess whether drug treatment might affect the expression of 14-3-3ζ, TAK-242, a selective TLR4 inhibitor [35] that blocks downstream inflammatory signaling, was administered to mini swine. Western blot analysis revealed that 14-3-3 zeta expression remained unaffected by TAK-242 treatment, reinforcing its reliability as a stable internal control under both pathological and treatment conditions.

## Conclusion

Overall, the Western blot analyses of key housekeeping proteins in swine RCT have revealed insightful expression profiles under both normal and hyperlipidemic conditions. The 14-3-3ζ protein emerged as a robust candidate, displaying consistent expression patterns independent of blood lipid levels and treatment conditions. This consistency underscores the reliability of 14-3-3ζ as a normalization control in Western blot analyses of tendon tissues in swine and provides valuable methodological insights for biomedical research, particularly in the study of musculoskeletal diseases and therapeutic interventions. Future studies may consider utilizing these insights to refine experimental approaches and improve the precision and reproducibility of protein expression in tendon tissues.

## Limitations of the study

While the small sample size in this study is a limitation, the findings strongly suggest 14-3-3ζ as a promising candidate for a housekeeping protein in swine rotator cuff tendon studies, demonstrating consistency across disease and treatment conditions. Future research should expand these investigations to include other types of tendon tissues to validate 14-3-3ζ as a reliable housekeeping gene in tendon research.

## Data Availability

All data with the actual images of the Western blot are provided within the manuscript.
